# Evaluation of the NIH Toolbox Cognition Battery across healthy agers and mild cognitive impairment among African‐American adults aged 65 and older

**DOI:** 10.1002/alz70861_108170

**Published:** 2025-12-23

**Authors:** Emily H Ho, Alec Easter, Berivan Ece, Bruno Giordani, Felicia C. Goldstein, Sandra Weintraub, Jennifer J. Manly, Richard C. Gershon

**Affiliations:** ^1^ Northwestern University Feinberg School of Medicine, Chicago, IL USA; ^2^ Northwestern University, Evanston, IL USA; ^3^ University of Michigan, Ann Arbor, MI USA; ^4^ Emory University School of Medicine, Atlanta, GA USA; ^5^ Mesulam Center for Cognitive Neurology & Alzheimer's Disease, Chicago, IL USA; ^6^ Department of Neurology, Columbia University, New York, NY USA; ^7^ Department of Neurology, Gertrude H. Sergievsky Center, Taub Institute for Research on Alzheimer's Disease and The Aging Brain, Columbia University Medical Center, New York, NY USA; ^8^ Northwestern University, Chicago, IL USA

## Abstract

**Background:**

Older African Americans are at increased risk compared with older White adults to have dementia of the Alzheimer’s type, yet few studies have investigated African‐American performance on standardized neuropsychological batteries such as the NIH Toolbox Cognition Battery (NIHTB‐CB). This study aimed to characterize the performance of NIHTB‐CB tests and composites in an African American sample of COGNITIVELY healthy agers and those diagnosed CLINICALLY with amnestic Mild Cognitive Impairment (aMCI).

**Methods:**

As part of the larger Advancing Reliable Measurement in Alzheimer’s Disease and Cognitive Aging (ARMADA) Study, we assessed 94 African‐American healthy agers (81.9% female; *M_age_* = 73.9, *SD_age_* = 5.06; 53.4% with 4‐year college degree or graduate‐level education) and 60 African‐American PARTICIPANTS with an MCI diagnosis (70% female; *M_age_* = 75.30, *SD_age_* = 5.94, 45% with higher education degree). The NIHTB‐CB was administered by trained examiners and completed a series of tests: the Crystallized Cognition Composite included Oral Reading Recognition (reading and pronunciation) and Picture Vocabulary (receptive vocabulary), and the Fluid Cognition Composite included List Sort Working Memory (LSWM; working memory), Picture Sequence Memory (PSM; episodic memory), Pattern Comparison Processing Speed (PC; processing speed), Dimensional Change Card Sort (DCCS; executive function/set‐shifting), Flanker Inhibitory Control and Attention (Flanker; executive function/inhibition/attention). In a series of ANOVAs using Bonferroni corrections, we investigated the differences in unadjusted scale scores, adjusting for age, education, sex as a biological variable, and study site. Partial eta‐squared (*η_p_^2^*) estimates were calculated to determine the effect size of the individual NIHTB‐CB tests/composites (*η_p_^2^* 0.01‐small, 0.06‐medium, 0.14‐large).

**Results:**

Compared to COGNITIVELY healthy agers, those with aMCI scored significantly lower on all NIHTB‐CB measures except for DCCS and PC. Large effect sizes were observed in particular for PV (*η_p_^2^* =.12), LSWM (*η_p_^2^* =.0.09), LSWM (*η_p_^2^* =.0.09), Flanker (*η_p_^2^* =.0.08), and PSM (*η_p_^2^* =.0.08). These differences were independent of sex or age effects, with the exception of LSWM and PSM, where an increase in age predicted lower performance.

**Conclusions:**

Nearly all measures within the NIHTB‐CB, including the Composite scores, significantly differentiate between healthy controls and individuals with aMCI among older African‐American adults. This is a highly educated sample, so true differences in the general population are likely of larger magnitudes.

